# Chromatin Structure in Telomere Dynamics

**DOI:** 10.3389/fonc.2013.00046

**Published:** 2013-03-07

**Authors:** Alessandra Galati, Emanuela Micheli, Stefano Cacchione

**Affiliations:** ^1^Dipartimento di Biologia e Biotecnologie, Istituto Pasteur – Fondazione Cenci Bolognetti, Sapienza Università di RomaRome, Italy

**Keywords:** telomere, telomeric chromatin, epigenetics, telomere dynamics

## Abstract

The establishment of a specific nucleoprotein structure, the telomere, is required to ensure the protection of chromosome ends from being recognized as DNA damage sites. Telomere shortening below a critical length triggers a DNA damage response that leads to replicative senescence. In normal human somatic cells, characterized by telomere shortening with each cell division, telomere uncapping is a regulated process associated with cell turnover. Nevertheless, telomere dysfunction has also been associated with genomic instability, cell transformation, and cancer. Despite the essential role telomeres play in chromosome protection and in tumorigenesis, our knowledge of the chromatin structure involved in telomere maintenance is still limited. Here we review the recent findings on chromatin modifications associated with the dynamic changes of telomeres from protected to deprotected state and their role in telomere functions.

## Introduction

The linear chromosomes of eukaryotes end with a specific nucleoprotein structure, named the telomere (Blackburn, 1991). In humans, telomeres consist of thousands of repeats of a six-base-pair motif (5′-TTAGGG-3′) ending in a G-rich 3′-overhang 30–300 nt long (Makarov et al., 1997), associated with a complex of six proteins named shelterin (de Lange, 2005). This structure prevents chromosome ends from being recognized as DNA double-strand breaks and therefore being processed by the DNA repair machinery. Due to the inability of DNA polymerases to completely replicate linear genomes – the so-called “end-replication problem” (Watson, 1972; Olovnikov, 2005) – cells undergo telomere shortening at every replication round. The loss of terminal DNA is counteracted by the activity of the telomerase enzyme, which adds *de novo* telomeric repeats to the G-rich 3′-overhang (Blackburn, 2005). In humans, telomerase is active only in germ line and in stem cells, but not in somatic cells where telomeres shorten till they reach a critical length that activates a DNA damage response (DDR) leading to replicative senescence or to apoptosis (Shay and Wright, 2005). This limit to the number of cell divisions constitutes an important barrier against cancer proliferation, because it reduces the risk of accumulating harmful mutations that could lead to malignant transformation. On the other hand, if cells escape p53 and Rb-dependent DNA damage checkpoints, telomere erosion could result in high chromosomal instability and facilitate the generation of tumor-promoting mutations (Artandi and DePinho, 2010). The re-activation of telomerase after these events stabilizes the genome and favors the establishment of malignant transformation. Besides its involvement in cancer, replicative senescence contributes to the physiology of aging and to the development of age-related diseases (Shay and Wright, 2005; Armanios and Blackburn, 2012).

Given the importance of telomeres in cancer establishment and in aging, the understanding of how protected and unprotected telomeres are structured is a very important issue in basic research. In this article we will examine the current knowledge regarding telomere structure and analyze the role of nucleosomes and chromatin organization in determining chromosome end-protection.

## The Dynamic Structure of Telomeres

Telomeres are extremely dynamic structures: in order to accomplish their multiple tasks their organization has to switch between a protected and a deprotected state throughout the cell cycle and cell differentiation (Blackburn, 2001).

During the cell cycle, telomere structure has to change from a closed conformation concealing chromosome ends from repairing enzymes, to an open one in S-phase in order to allow controlled access to DNA replication factors. A dramatic change occurs in telomerase negative cells as a consequence of telomere shortening. When they reach a critical short length, telomeres uncap triggering an ataxia telangiectasia mutated (ATM) and/or ataxia telangiectasia and Rad3 related (ATR) signaling cascade (d’Adda di Fagagna et al., 2003), that eventually leads to a p53-dependent cell cycle arrest or to apoptosis (Harley et al., 1990; Herbig et al., 2004).

The nature of telomere structure in protected and deprotected states has not been completely clarified. Capping requires the binding of specific proteins that recognize telomeric DNA and shield single-stranded G-overhangs by hiding them into specific structures. An attractive solution, although not necessarily exclusive, to the end capping dilemma is represented by the telomeric loop, or t-loop (de Lange, 2004). T-loops have been identified by electron microscopy visualizations of purified telomeric DNA treated with the cross-linking agent psoralen (Griffith et al., 1999). In these lasso-like structures, the 3′-overhang folds back and invades the upstream telomeric region, generating a displacement loop (D-loop). T-loops are very variable in size and have been found in several eukaryotes (Tomaska et al., 2004), although it is not clear whether they are present at all telomeres (de Lange, 2004). An alternative structure capable of sheltering the free chromosome ends is a four-stranded DNA structure named G-quadruplex (Sen and Gilbert, 1988), which derives from the folding of single-stranded DNA containing runs of three to four consecutive guanines to form stacked tetrads of Gs, stabilized by Hoogsteen hydrogen bonding and cation coordination. Although *in vitro* telomeric G-rich single-stranded DNAs form very stable G-quadruplex structures easily, their existence *in vivo* is controversial (Lipps and Rhodes, 2009). Several proteins are able to bind to, cleave, resolve, or promote the formation of telomeric G-quadruplexes from several species *in vitro* (Oganesian and Bryan, 2007). In addition, a synthetic small molecule was shown to mediate the selective isolation of human telomeric DNA, containing G-quadruplex motifs, from human cells (Muller et al., 2010). The first evidence of the *in vivo* formation of G-quadruplexes has been obtained in ciliates (Paeschke et al., 2005), by using specific antibodies against the G-quadruplex structure. In mammals, indirect proofs of G-quadruplex formation came from several studies that used G-quadruplex stabilizing molecules as telomerase inhibitors in anticancer strategies (De Cian et al., 2008; Micheli et al., 2009; Bryan and Baumann, 2011). Very recently, using a highly specific DNA G-quadruplex antibody, these structures have been visualized in human cells (Biffi et al., 2013). Interestingly, the formation of G-quadruplexes increased during S-phase. T-loop and G-quadruplex represent two possible solutions to the end-protection problem and could embody different functional states at telomeres. Interestingly, both structures need the activity of the RTEL1 helicase in order to be resolved and allow an efficient telomere replication (Vannier et al., 2012).

However, other structural solutions to the end-protection problem have been recently described. 5′-C-overhangs have been recently discovered in *Caenorhabditis elegans*, where they are as abundant as G-overhangs (Raices et al., 2008), and have been also found in mouse and human cells (Oganesian and Karlseder, 2011). C-overhangs are present at low frequency in primary cells, whereas they are enriched in cells that have established a telomerase-independent mechanism of telomere maintenance named ALT (alternative lengthening of telomeres), based on recombination (Bryan et al., 1997). Another structural solution has been recently identified in *Arabidopsis thaliana*. In this plant, telomeres deriving from leading strand replication are not processed to generate 3′ G-overhangs, but are conserved as blunt-ended telomeres (Kazda et al., 2012). Their protection depends on the Ku70/Ku80 heterodimer, a complex involved in DNA repair activities. Interestingly, Ku-depleted telomeres are still functional, suggesting the existence of several alternative structures able to protect chromosome ends.

## Shelterin and Friends: A Lot of Players at the End of Chromosomes

Telomeric DNA interacts with several factors to accomplish its tasks. Extensive research has been devoted to understand the functions carried out by the specific proteins that form the protective complex at telomeres, known in mammals as the shelterin complex. Less clear is the role played by nucleosomes, although they organize most of telomeric chromatin. Also the functions of TERRA, the recently discovered non-coding RNAs transcribed at the telomeres, are not yet understood.

### Shelterin

In mammals, the shelterin complex is composed of six proteins, TTAGGG repeat binding factors 1 and 2 (TRF1, TRF2), protection of telomeres 1 (POT1), repressor/activator protein 1 (RAP1), TRF1-interacting nuclear factor 2 (TIN2), and TIN2-interacting protein 1 (TPP1) (for a review, see Palm and de Lange, 2008; Diotti and Loayza, 2011). Double-stranded telomeric repeats are bound by TRF1 and TRF2, whereas the single-stranded overhang is bound by POT1, which forms a heterodimer with TPP1. RAP1 interacts in a 1:1 ratio with TRF2; the sixth member of shelterin, TIN2 connects TRF1 and TRF2 with the heterodimer TPP1/POT1. Shelterin acts by inhibiting DDR pathways at telomeres. Deletion of each individual shelterin protein causes destabilization of the capping structure in a way that resembles spontaneous telomere deprotection consequent to extreme telomere erosion. Even if the shelterin complex represents a functional unit, specific functions can be attributed to individual shelterin components. The ATM kinase pathway is repressed by TRF2 (Celli and de Lange, 2005; Denchi and de Lange, 2007). TRF2 depletion results in the accumulation at telomeres of DDR factors such as γ-H2AX, 53BP1, mediator of DNA damage checkpoint 1 (MDC1) (Takai et al., 2003) forming DNA damage foci called TIFs (Telomere dysfunction-Induced DNA damage Foci). Instead, activation of the ATR signaling pathway requires the removal of POT1 (Denchi and de Lange, 2007). TRF2 and POT1 are also key players in preventing two major DNA repair activities, Non-Homologous End-Joining (NHEJ) and Homology Directed Repair (HDR) (de Lange, 2009). Removal of the entire shelterin complex from mouse telomeres revealed protection from two other DDR pathways, alternative NHEJ (alt-NHEJ) and resection (Sfeir and de Lange, 2012).

The shelterin proteins are also involved, either directly or indirectly by recruiting accessory factors, in regulating telomerase activity and access to chromosome ends (Wang et al., 2007; Baumann and Price, 2010), allowing the correct replication of the telomeric duplex (Sfeir et al., 2009), promoting the generation and maintenance of G-overhangs (Zhu et al., 2003; Wu et al., 2010), affecting telomere topology (Amiard et al., 2007), and favoring t-loop formation (Stansel et al., 2001; Poulet et al., 2009). A list of proteins that interact with shelterin and their known function at telomeres is reported in Table 1.

**Table 1:** **Proteins that interact with telomeric DNA and shelterin components**.

Protein	Org.	Binding partners	Function(s)	Reference
CST	h	Tel-DNA, POT1-TPP1	Inhibition of telomerase activity	Chen et al. (2012)
MRE11, RAD50, NBS1	h, m	TRF2, TRF1	DDR; 3′-overhang generation	Wu et al. (2000, 2007a), Zhu et al. (2000), Misri et al. (2008), Deng et al. (2009a)
ATM	h	TRF2	Role in DDR	Karlseder et al. (2004)
Rad9	h	TRF2, Rad51	DDR; role in HR	Pandita et al. (2006)
WRN	h	Tel-DNA, TRF1, TRF2, POT1, Ku70/80, BLM	DNA replication; resolution of secondary structures; inhibition of telomere circle formation	Opresko et al. (2004, 2005), Li et al. (2008)
FEN1	h	TRF2, WRN	Telomere replication	Saharia et al. (2008, 2010)
PINX1	h	TERT; recruited by TRF1	Inhibition of telomerase activity	Zhou and Lu (2001)
GNL3L	h	TRF1	TRF1 homodimerization and stabilization	Zhu et al. (2009)
NS	m, h	TRF1	TRF1 turnover	Zhu et al. (2006)
PIN1	h, m	TRF1 (phosphoT149)	TRF1 turnover	Lee et al. (2009)
Fbx4	h	TRF1	TRF1 turnover	Lee et al. (2006)
Apollo	h, m	Tel-DNA; recruited by TRF2	3′-Overhang maintenance	Freibaum and Counter (2006), Lenain et al. (2006), Lam et al. (2010), Wu et al. (2010)
PNUTS	h	Recruited by TRF2	Possible role in DDR	Kim et al. (2009), Landsverk et al. (2010)
MCPH1 (BRIT1)	h	Recruited by TRF2	Possible role in DDR	Kim et al. (2009), Lin et al. (2010)
Ku70/80	h, m	TRF1, TRF2	Telomere protection from deletions and HDR	Hsu et al. (2000), Celli et al. (2006), Wang et al. (2009)
DNA-PKcs	m	Tel-DNA	Telomere capping	d’Adda di Fagagna et al. (2001), Gilley et al. (2001)
ATRX	h	Tel-DNA	Incorporation of H3.3 at telomeres	Law et al. (2010)
CSB	h	TRF2	Telomere stability; TERRA homeostasis	Batenburg et al. (2012)
TRIP6, LPP	h	TRF2, TIN2, probably POT1	Possible DDR inhibition	Sheppard and Loayza (2010)
HP1γ	h	TIN2	Maintenance of telomere cohesion	Canudas et al. (2011)
RTEL1	m	nd	T-loop disassembling; G4-DNA unwinding; prevention of telomere fragility	Vannier et al. (2012)
BLM	h	TRF1, TRF2, WRN	G4-DNA unwinding; resolution of late-replicating structures	Sun et al. (1998), von Kobbe et al. (2002), Barefield and Karlseder (2012)
SIRT1	m	Tel-DNA, Nbs1, WRN	Promotion of HR; TL	Palacios et al. (2010)
Tankyrase 1	h	TRF1, DNA-PKcs	T-SCE suppression and DNA-PKcs stabilization	Dregalla et al. (2010)

*org, organism; h, human; m, mouse; nd, not determined; tel-DNA, telomeric DNA; DDR, DNA damage response; HR, homologous recombination; NHEJ, non-homologous end-joining; HDR, homology directed repair; TERRA, telomeric repeat containing RNA; G4-DNA, G-quadruplex DNA; TL, telomere lengthening; T-SCE, telomere sister chromatid exchanges*.

Since telomeric sequences are highly evolutionarily conserved, it is not surprising that shelterin homologs have been characterized in several species. In fission yeast, double-stranded telomeric DNA is bound by TAZ1, a TRF1/TRF2 ortholog (Cooper et al., 1997), whereas the single-stranded binding protein POT1 forms a complex with TPZ1, a homolog of TPP1 (Miyoshi et al., 2008). In *Saccharomyces cerevisiae*, RAP1 binds directly to the telomeric double-stranded repeats (Gilson et al., 1993), whereas the CST (Cdc13/Stn1/Ten1) complex binds the single-stranded DNA (for a review of yeast telomeres; Wellinger and Zakian, 2012). *Drosophila* represents an exception to the evolutionarily conserved organization of telomeres. In *Drosophila* telomerase is absent and telomeres consist of transposable elements (Mason et al., 2008); end capping is independent from DNA sequence and is assured by the protein complex named terminin, that includes at least four proteins: HOAP, HipHop, Modigliani (Moi), and Verrocchio (Ver) (Raffa et al., 2011). Terminin proteins are not conserved outside the Drosophilidae, with the exception of Ver, which exhibits structural homology with Stn1 (Raffa et al., 2010).

### Telomeric RNA

Since their discovery and characterization, telomeres have been considered transcriptionally silent chromosomal regions. The spreading of the heterochromatic state from the telomeres toward the subtelomeric regions causes transcriptional repression of nearby genes or telomere position effect (TPE) (Gottschling et al., 1990; Koering et al., 2002; Ottaviani et al., 2008). This concept changed with the discovery that telomeres are transcribed into long non-coding RNAs (Azzalin et al., 2007). These telomeric repeat containing RNAs, named TERRA, have been found in several eukaryotic species, from yeast to mammals (Schoeftner and Blasco, 2008; Bah et al., 2012; Greenwood and Cooper, 2012). TERRA are transcribed mainly by RNA Polymerase II, starting from subtelomeric promoters that have been partially identified in human cells (Nergadze et al., 2009). The role of TERRA in telomere capping remains elusive. In yeast TERRA negatively affects telomere length (Maicher et al., 2012; Pfeiffer and Lingner, 2012). A relationship between TERRA transcription and telomere length has been found also in human cells, where TERRA levels seem to be negatively regulated by telomere elongation through the increase of heterochromatic marks at telomeres (Arnoult et al., 2012). Although *in vitro* experiments suggest that TERRA levels negatively affect telomerase activity (Redon et al., 2010), recent findings show that telomere homeostasis is independent of telomere transcription (Farnung et al., 2012).

### Telomeric nucleosomes

Similarly to the rest of chromosomal DNA, the long telomeres of higher eukaryotes are organized in nucleosomes (Pisano et al., 2008). Nucleosomes derive from the wrapping of 147 bp of DNA around a globular basic protein complex, the histone octamer. Not only nucleosomes represent a way for eukaryotes to pack their abundant DNA content in their small nuclear volume, but they are also a key factor in the regulation of several biological processes (Luger, 2006). Most telomeric DNA is organized in an unusual chromatin structure, characterized by regularly spaced and tightly packed nucleosomes separated by DNA linkers about 40 bp shorter than in bulk DNA (Makarov et al., 1993; Tommerup et al., 1994; Fajkus et al., 1995; Lejnine et al., 1995). This peculiar organization is likely to reflect both the interplay with telomeric proteins and the intrinsic properties of telomeric sequences. DNA sequence plays an important role in determining several features of nucleosomes, such as thermodynamic stability and positioning, dictating which DNA tracts directly interact with the histone octamer. The wrapping of DNA around the histone octamer is ruled by intrinsic features such as DNA flexibility and stiffness (Anselmi et al., 2000; Filesi et al., 2000). Since telomeric repeats (mostly 5–8 bp long) are out of phase with the DNA helical repeat in the nucleosome (10.2 bp), it has been suggested that telomeric DNAs may require more energy that the remaining genomic DNA to wind around the histone octamer (Fajkus et al., 1995; Pisano et al., 2008). In line with this hypothesis, *in vitro* reconstitution studies showed that telomeric DNAs form the least stable nucleosomes among all the DNA sequences studied so far (Cacchione et al., 1997; Rossetti et al., 1998; Filesi et al., 2000). Moreover, telomeric nucleosomes can occupy multiple isoenergetic positions (Rossetti et al., 1998; Filesi et al., 2000), i.e., they form with the same probability on several positions along telomeric DNA. The lack of positioning signals implies that the energy required for the histone octamer to slide from one position to another along the telomeric DNA is low (Filesi et al., 2000); therefore, telomeric nucleosomes are highly intrinsically mobile *in vitro* at physiological ionic strengths, as shown by atomic force microscopy (AFM) imaging (Pisano et al., 2007). Sequence-dependent features are also a major determinant of nucleosome spacing at telomeres. AFM visualizations of *in vitro* assembled nucleosomal arrays demonstrated that at low histone octamer/DNA ratios (about one nucleosome every 300–400 bp) the spacing between telomeric nucleosomes is irregular, whereas at saturating histone/DNA ratios the inter-nucleosomal distance is comparable to the value found *in vivo* (Mechelli et al., 2004; Pisano et al., 2006). A regular and short nucleosomal spacing has been reported also by *in vitro* assembly at near-physiological conditions (Galati et al., 2012). In their experiments the authors used *Drosophila* embryonic extracts to assemble a nucleosomal array in the presence of histone chaperones and chromatin remodeling complexes. Whereas the nucleosomal repeat length is about 200 bp in arrays reconstituted on different control DNAs, in the case of telomeric DNA the repeat length was about 160 bp, strongly indicating that the short nucleosomal spacing is an intrinsic feature of telomeric sequences (Galati et al., 2012).

Little is known about the higher order organization of telomeric chromatin. The linker histone H1, normally present at 1:1 H1/histone octamer ratio in bulk chromatin, seems to be under-represented at telomeres (Bedoyan et al., 1996; Dejardin and Kingston, 2009). Moreover, the short nucleosomal spacing suggests that higher order chromatin condensation would be peculiar. Different models have been proposed, ranging from a narrow columnar packaging (Fajkus and Trifonov, 2001) to a nucleosomal folding in which diameter of the fiber (ranging from 25 to 30 nm) and angles between nucleosomes are dictated by the length of linker DNA (Besker et al., 2003). Consistent with the latter model, electron microscopy images of chromatin fractions enriched in telomeric chromatin from differentiated tissues such as chicken erythrocytes and mouse lymphocytes, showed that telomeres are organized in a fiber of about 30 nm diameter (Nikitina and Woodcock, 2004).

## A Telomeric Histone Code?

Histone post-translational modifications and histone variants play a key role in the regulation of most cellular processes, including transcription, DNA repair, and recombination. Numerous post-translational modifications have been found associated with telomeric regions (reported in Table 2), and various factors have been shown to influence the organization of telomeric chromatin (see Table 3). Telomeric chromatin has been generally considered as “heterochromatic,” mainly on the basis of extensive studies on yeast and *Drosophila* telomeres, in which the establishment of a heterochromatic state at telomeres and subtelomeres is essential for the protection of chromosome ends (Shore, 2001; Raffa et al., 2011). In budding yeast telomeres are short and form a nucleosome-free structure (Wright et al., 1992). Telomeric double-stranded repeats are bound by the protein RAP1 which recruits among other proteins the Sir complex [Silent Information Regulators, Sir2 (a histone deacetylase), Sir3, and Sir4]. The Sir complex is essential for the formation of a heterochromatic complex that spreads in the subtelomeric region, giving rise to the repression of nearby genes (Ottaviani et al., 2008).

**Table 2:** **Post-translational modifications (PTM) at telomeres**.

Modification target	Org.	PTM type	Responsible enzyme	Function(s)	Reference
H3K9	m	Methylation	SUV39H1, SUV39H2	Chromatin compaction	Blasco (2007)
H4K20	m	Methylation	SUV4-20H1, SUV4-20H2	Chromatin compaction	Blasco (2007)
H3K79	m	Methylation	Dot1L	Chromatin compaction	Jones et al. (2008)
H2BK5, H3K4	h	Methylation	nd	nd	Rosenfeld et al. (2009)
H3K9	At	Methylation	nd	nd	Vrbsky et al. (2010)
H3K27, H3K4	At	Methylation	nd	nd	Vrbsky et al. (2010), Vaquero-Sedas et al. (2012)
H2AX	h	Phosphorylation	ATM, ATR, DNA-PKcs	DDR signaling	Takai et al. (2003)
H2A, H2AX	m	Ubiquitylation	RNF8	DDR signaling	Peuscher and Jacobs (2011), Jacobs (2012)
H2A, H2AX	m, h	Ubiquitylation	RNF168	Recruitment of 53BP1; NHEJ signaling	Doil et al. (2009), Okamoto et al. (2013)
H4K12	Sc	Acetylation	NuA4	Regulation of chromatin plasticity and accessibility	Zhou et al. (2011)
H3K9	At	Acetylation	nd	nd	Vaquero-Sedas et al. (2012)
H3K9ac, H3K56ac	h	Deacetylation	SIRT6	Chromatin compaction	Michishita et al. (2008), reviewed in Tennen and Chua (2011)
H3K9ac	m	Deacetylation	SIRT1	Chromatin compaction	Palacios et al. (2010)
TRF1	h	Ubiquitylation	Fbx4, RLIM	TRF1 turnover	Chang et al. (2003), Her and Chung (2009)
TPP1	m, h	Ubiquitylation	RNF8	TPP1 stabilization; A-NHEJ repression	Rai et al. (2011)
TRF2	h	Ubiquitylation	Siah1	TRF1 turnover, induced by p53	Fujita et al. (2010)
TIN2*	h	Ubiquitylation	Siah2	TIN2 depletion; possible role in shelterin remodeling	Bhanot and Smith (2012)
TRF1	h	Poly-ADP-ribosylation	Tankyrase (1 and 2)	Decrease of TRF1 affinity for the DNA; TRF1 proteasomal degradation; TL	Smith and de Lange (2000), Cook et al. (2002), Dynek and Smith (2004)
Ub-TRF1	m, h	Deubiquitylation	USP22	TRF1 turnover; stabilization of WRN telomere association	Atanassov et al. (2009)
TRF1, TRF2	m, h	Sumoylation	SMC5/6	HR and TL (in ALT cells)	Potts and Yu (2007)
TRF2	h	Methylation	PRMT1	Telomere stability; telomere length regulation	Mitchell et al. (2009)
TRF2	h	Phosphorylation	Aurora C*, ATM, CHK2	Decrease of TRF2 binding to telomere	Tanaka et al. (2005), Spengler (2007), Buscemi et al. (2009)
TRF1	h	Phosphorylation	Plk1, CK2	Increase of telomere binding; role in TRF1 expression-induced apoptosis; TS	Kim et al. (2008), Wu et al. (2008)
TRF1	h	Phosphorylation	ATM, Cdk1	Decrease of TRF1 binding to telomere; TL	Wu et al. (2007a), McKerlie and Zhu (2011), McKerlie et al. (2012)
TRF1	h	Phosphorylation	Akt*	TS	Chen et al. (2009)
Cdc13	Sc	Sumoylation	Siz1, Siz2	Telomerase inhibition	Hang et al. (2011)
Yku70/80, Sir4	Sc	Sumoylation	Siz2	Anchoring at NE, preventing TL	Ferreira et al. (2011)
TERT	h	Ubiquitylation	MKRN1, Hdm2, CHIP	TERT proteasomal degradation; TS	Kim et al. (2005), Lee et al. (2010), Oh et al. (2010)
TERT	h	Phosphorylation	Tyrosine kinase c-Abl	Inhibition of TERT activity; TS	Kharbanda et al. (2000)
Subtelomeric DNA	m	Methylation	DNMT1, DNMT3a, DNMT3b	Chromatin compaction; recombination repression; TS	Gonzalo et al. (2006)
nd	m	Phosphorylation	DNA-PKcs	Telomere protection	Bailey et al. (2004), Williams et al. (2009)
nd	Sc	Ubiquitylation	Cul8	Transcriptional silencing	Mimura et al. (2010)

org, organism; m, mouse; h, human; At, *Arabidopsis thaliana*; Sc, *Saccharomyces cerevisiae*; nd, not determined; *, *in vitro* evidences; DDR, DNA damage response; A-NHEJ, alternative non-homologous end-joining; TL, telomere lengthening; HR, homologous recombination; TS, telomere shortening; NE, nuclear envelope.

**Table 3:** **Factors influencing telomeric chromatin**.

Name	Org.	Binding partners	Function(s)	Reference
ORC	h	Tel-DNA, TERRA; TRF2	DNA replication; inhibition of telomere circle formation	Deng et al. (2007, 2009b)
TERRA	h	TRF1, TRF2, ORC. HP1, H3K9me3	Facilitation of TRF2-ORC interaction; chromatin compaction	Deng et al. (2009b)
ERCC1/XPF	h, m	Tel-DNA	3′-Overhang degradation (in uncapped telomeres)	Zhu et al. (2003), Munoz et al. (2005), Wu et al. (2007b)
53BP1	m	H4K20me2	Promotion of NHEJ; increase of telomere mobility (in dysfunctional telomeres)	Dimitrova et al. (2008)
SHREC	Sp	Ccq1	Regulation of nucleosome positioning; telomeric silencing	Sugiyama et al. (2007)
14-3-3σ	h	nd	Help in DDR (G2 arrest)	Dhar et al. (2000)
DNA-Pkcs	h, m	WRN	B-NHEJ repression; promotion of WRN helicase activity on D-loop; capping	Gilley et al. (2001), Bombarde et al. (2010), Kusumoto-Matsuo et al. (2010)
HP1	h, d, m	H3K9me3, tel-DNA (d)	Transcription repression; capping; chromatin compaction	Lachner et al. (2001), Koering et al. (2002), Garcia-Cao et al. (2004), Perrini et al. (2004)
Cbx1, Cbx3, Cbx5	m	H3K9me3	Chromatin compaction	Garcia-Cao et al. (2004)
Daxx/ATRX	m	H3.3	Deposition of H3.3	Goldberg et al. (2010), Lewis et al. (2010)
ATRX	h, m	mH2A	Inhibition of mH2A1 incorporation; TERRA repression (m)	Ratnakumar et al. (2012)
Rb1, Rbl1, Rbl2	m	HP1, SUV4-20H HMTases	TS and chromatin compaction	Garcia-Cao et al. (2002), Gonzalo and Blasco (2005)
miR-290 (Dicer-dependent)	m	Rbl2 mRNA	Regulation of DNA methylation	Benetti et al. (2008a), Sinkkonen et al. (2008)

*org, organism; h, human; m, mouse; Sp, *Schizosaccharomyces pombe*; d, *Drosophila melanogaster*; nd, not determined; tel-DNA, telomeric DNA; TERRA, telomeric repeat-containing RNA; NHEJ, non-homologous end-joining (B, backup; C, classical); DDR, DNA damage response; TS, telomere shortening*.

*Drosophila* telomeres are enriched in histone marks such as trimethylation of Lys 9 of the H3 histone (H3K9me3). This histone mark is recognized by heterochromatin protein 1 (HP1), an essential factor for the protection of *Drosophila* telomeres (Fanti et al., 1998) and for the spreading of heterochromatin through the recruitment of the histone methyltransferase (HMTase) SU-VAR3-9 (Schotta et al., 2002).

Heterochromatin formation has an important function also for telomere capping in *Schizosaccharomyces pombe*. In this organism, telomere protection is assured by a complex of shelterin-like proteins (Moser and Nakamura, 2009). In the absence of telomerase, *S. pombe* cells can survive telomere loss by adopting an alternative mode to protect chromosome ends based on amplification and rearrangement of heterochromatic regions (Jain et al., 2010). In these survivors, named HAATI (heterochromatin amplification-mediated and telomerase-independent), the telomeric 3′-overhang still binds the protein POT1 and its interacting partner Ccq1 (Miyoshi et al., 2008), which is known to bind also the heterochromatic complex Snf2/HDAC-containing repressor complex (SHREC) (Sugiyama et al., 2007). These data suggest that heterochromatin could recruit POT1 to telomeres via the Ccq1/SHREC complex, allowing POT1 binding to chromosome ends even in the absence of its specific recognition sequences (Jain et al., 2010). These particular telomeres in *S. pombe* resemble *Drosophila* telomeres, that lack specific sequence recognition by telomeric proteins (Raffa et al., 2011).

### Epigenetic state of telomeres in plants and mammals

The epigenetic state of organisms with long telomeres such as higher plants and mammals is much less definite. This is partly due to the difficulty of obtaining clear results from ChIP analyses. The hybridization with a telomeric probe does not allow distinguishing between real terminal telomeric sequences and telomeric repeats located at internal sites on the genome, the so-called internal telomeric sequences (ITSs). ITSs are present at subtelomeric and internal positions in several plants and vertebrates, including humans (Meyne et al., 1990; Azzalin et al., 2001; Lin and Yan, 2008). Analyses of histone marks at ITS and at telomeres have been recently carried out in *Arabidopsis* by two different research groups. The first group used stringent hybridization conditions to distinguish ITSs from telomeres and found the presence of both heterochromatic and euchromatic marks at *Arabidopsis* telomeres and subtelomeres (Vrbsky et al., 2010). The second group used the restriction enzyme *Tru*9I to distinguish between ITS and telomeric repeats. *Tru*9I cuts most interspersed ITSs with a recognition motif 5′-TTAA-3′, leaving intact the telomeric 5′-TTTAGGG-3′ repeats. *Arabidopsis* subtelomeric regions and ITSs result enriched in heterochromatic marks whereas telomeres exhibit euchromatic features such as H3K9 and H4K16 acetylation (Vaquero-Sedas et al., 2011). A later work by the same research group addressed the epigenetic state of *Arabidopsis* telomeres and centromeres by ChIP-sequence analysis (Vaquero-Sedas et al., 2012), substantially confirming the presence of some euchromatic marks at telomeres. In addition, both subtelomeric and telomeric DNA were methylated in *Arabidopsis* and tobacco (Cokus et al., 2008; Vrbsky et al., 2010; Majerova et al., 2011). Altogether, these studies showed a mix of both euchromatic and heterochromatic marks at plant telomeres.

In mammals, extensive studies have been carried out in mouse to characterize the epigenetic marks associated with telomeres and subtelomeres (Blasco, 2007). ChIP analyses have demonstrated that both regions were enriched in heterochromatic marks, namely H3K9me3 and H4K20me3, and were hypoacetylated in H3 and H4; in addition, subtelomeric DNA was heavily methylated. The enrichment at telomeres of H3K9me3 and H4K20me3 was confirmed by genome-wide mapping of the chromatin state of mouse embryonic stem (ES) cells, neural progenitor cells, and embryonic fibroblasts (Mikkelsen et al., 2007). H3K9me3 is responsible for the recruitment at telomeres of HP1 proteins (HP1α, HP1β, and HP1γ) through a high affinity binding site (Lachner et al., 2001). Similarly to *Drosophila*, HP1 mouse isoforms promote the spreading of heterochromatin by interacting with the HMTases SUV4-20H1/2 that catalyzes the trimethylation of H4K20 (Schotta et al., 2004). The establishment of a heterochromatic region is important for the structural integrity of mouse telomeres; knockout deletions of HMTases (SUV39H1/2, SUV4-20H1/2) and DNA methyltransferases (DNMT3A/B, and DNMT1) result in defective telomere function, aberrantly increased telomere length, and chromosomal instability (Garcia-Cao et al., 2004; Gonzalo et al., 2005, 2006). Also the methylation of another lysine of the H3 histone, H3K79, by the methyltransferase Dot1, is required for the formation of a heterochromatic telomere structure (Jones et al., 2008). Consistent with these observations, deficiency of the deacetylase Sirt1, a mouse ortholog of yeast Sir2, causes a decrease of heterochromatic marks and triggers a DDR at telomeres (Palacios et al., 2010).

### Are human telomeres heterochromatic?

Less clear is the epigenetic state of human telomeres. ChIP experiments indicate that the levels of heterochromatic marks such as H3K9me3, H4K20me3, and H3K27me3 are unexpectedly low at telomeres in human fibroblasts (O’Sullivan et al., 2010). A ChIP-seq analysis of histone marks at non-coding regions of human CD4^+^ T-cells showed that telomeres are significantly enriched in H2BK5me1 and H3K4me3, two post-translational histone modifications often found associated with actively transcribed genes (Rosenfeld et al., 2009). H3K9me3 is under-represented at telomeres, whereas it is enriched at subtelomeres, although at lower levels than at centromeric and pericentromeric regions (Rosenfeld et al., 2009). Another genome-wide analysis addressed the chromatin profile of nine different human lines (Ernst et al., 2011). In this study, the chromatin state of repetitive sequences (therefore not only telomeric DNA) resulted enriched in heterochromatic marks such as H3K9me3. However, direct evidence that the establishment of a heterochromatic state at human telomeres is important for chromosome stability was derived from studies analyzing the effects of the depletion of SIRT6, a member of the Sir2 family (also called sirtuins). In yeast, Sir2 promotes the transcriptional silencing at several heterochromatic regions including subtelomeres (Gottschling et al., 1990), by deacetylating several lysines on N-terminal histone tails (Imai et al., 2000). SIRT6 is a Nad^+^-dependent histone deacetylase that specifically removes acetyl residues from H3K9 (Michishita et al., 2008) and H3K56 (Michishita et al., 2009) at telomeres. Knockout of human Sirt6 leads to hyperacetylation of H3K9 and H3K56, and has severe consequences on chromosome stability, resulting in telomere fusions and premature senescence (Michishita et al., 2008), and in the abrogation of the TPE (Tennen et al., 2011). These data suggest that the presence of histone marks generally associated with a heterochromatic state, such as hypoacetylation, is essential for the protective capping of human telomeres. In agreement with this, recent data showed that the heterochromatic protein HP1-γ binds the C-terminal domain of TIN2 and is required for telomere cohesion during S-phase (Canudas et al., 2011).

Another layer of complexity and flexibility is added by the presence of histone variants, non-allelic isoforms of the four canonical histones H2A, H2B, H3, and H4. Histone variants genes are generally present in single copy in the genome, and are expressed throughout the cell cycle, whereas canonical histone genes are clustered in repeated arrays and are almost exclusively transcribed during the S-phase. Recently, it has been shown that mouse and human telomeres contain the histone H3 variant H3.3 (Goldberg et al., 2010; Ratnakumar et al., 2012). H3.3 is enriched within actively transcribed genes, through a replication-independent deposition mechanism catalyzed by the histone chaperone HIRA. Instead, H3.3 deposition at telomeres is mediated by the remodeling complex ATRX in cooperation with the histone chaperone DAXX. Knockdown of ATRX by RNAi causes telomere dysfunctions in mouse ES cells (Wong et al., 2010) and up-regulation of TERRA expression (Goldberg et al., 2010). Interestingly, ATRX is a negative regulator of the deposition of macroH2A, a H2A variant abundant in heterochromatic domains such as the inactivated X chromosome (Ratnakumar et al., 2012). Recently, ATRX and DAXX mutations have been associated with pancreatic neuroendocrine tumors (Heaphy et al., 2011; Jiao et al., 2011) and pediatric glioblastoma (Schwartzentruber et al., 2012) and with the establishment of a ALT mechanism of telomere maintenance (Lovejoy et al., 2012).

The emerging view is that human telomeres are characterized by a mix of heterochromatic and euchromatic marks, which give rise to a specific epigenetic pattern with functions and implications that need to be further elucidated.

### Chromatin modifications in deprotected telomeres

The deprotection of the telomeric capped structure, either as a consequence of telomere shortening or due to shelterin dysfunction, leads to dramatic changes that affect also the epigenetic pattern and the nucleosomal organization of telomeres. Experiments in telomerase negative mice (*terc*^−^/^−^) showed that telomere shortening correlates with a significant decrease of two heterochromatic marks at telomeres and subtelomeres, H3K9me3 and H4K20me3, and with the increase of H3 and H4 acetylation (Benetti et al., 2007). Moreover, there is a decrease of subtelomeric DNA methylation and of the binding of CBX3 (homolog of the *Drosophila* protein HP1) to telomeres and subtelomeres.

Several histone modifications are associated with the DDR consequent to shelterin loss, or to extreme telomere shortening. The activation of ATM and/or ATR signaling leads to the phosphorylation of the histone variant H2AX (γ-H2AX) on its C-terminal tail (Miller and Jackson, 2012). This modification is not limited to DNA damage at telomeres, since it represents one of the most evident modifications following DNA DSBs along the genome, encompassing an area of about 1 Mb around the site of DNA damage (Iacovoni et al., 2010). γ-H2AX acts recruiting MDC1 (Stucki et al., 2005) and other factors at the DBS. ATM-phosphorylated MDC1 in turn recruits RNF8 (Ring Finger Protein 8), an E3 ubiquitin ligase that cooperates with the E2 conjugating enzyme UBC13 to ubiquitylate histones H2A and H2AX (Mailand et al., 2007). A second Ring type E3 ubiquitin ligase enzyme, RNF168, binds ubiquitinylated histones and catalyzes the spreading of histone ubiquitylation around the DNA damage site (Doil et al., 2009), mediating the recruitment of 53BP1, which in turn promotes telomere fusions. At mammalian telomeres, RNF8 acts on multiple targets. Besides playing a key role in mediating telomeric DDR, evidenced by its accumulation at uncapped telomeres and by favoring telomere fusions (Peuscher and Jacobs, 2011), RNF8 counteracts telomere dysfunction by binding and stabilizing TPP1 at telomeres (Rai et al., 2011). The recruitment of RNF168 is inhibited by the activities of the deubiquitinating enzyme BRCC3, which counteract the action of RNF8, and of the ubiquitin ligase UBR5, which targets RNF168 for degradation. At telomeres, these two enzymes are recruited by TRF2 through the C-terminal region of the hinge domain, named inhibitor of DDR (iDDR) (Okamoto et al., 2013).

The DNA damage signaling consequent to telomere shortening seems to cause a global chromatin change. Upon aging of human fibroblasts, the synthesis of histones decreases, as well as the synthesis of the histone chaperones ASF1 and CAF1, and of stem loop binding protein (SLBP), a stabilizer of histone mRNA (O’Sullivan et al., 2010). Moreover, the global and the telomere-specific distribution of histone marks throughout the cell cycle are altered as a consequence of cellular aging. Re-activation of telomerase is sufficient to revert these changes (O’Sullivan et al., 2010).

## Crosstalk Between Shelterin, Histones, and Terra at Telomeres

In the dynamic change from a protected to a deprotected telomeric state, a relevant issue concerns the interplay between the actors present at telomeres. Telomere shortening causes a reduction in telomeric repeats; therefore, it is easy to predict an increased competition between TRF1, TRF2, and the histone octamer for binding to telomeric DNA. Little is known about the relative abundance of these proteins at telomeres and whether it varies with changes in telomere length. De Lange and coworkers measured the stoichiometry of the six proteins forming the shelterin complex in different human cell lines (Takai et al., 2010). Assuming that all the shelterin proteins in the chromatin-bound fraction are associated with telomeres, the authors estimated their abundance at telomeres. Although the absolute number of chromatin-bound molecules seems variable and independent from telomere length, the densities of TRF1 and TRF2 increase at short telomeres (Takai et al., 2010) and their abundance is sufficient to saturate the entire telomeres. However, TRF1 and TRF2 have to recognize their binding sites in a dense nucleosomal context. Even if a measurement of histone absolute density at telomeres is lacking, data from MNase digestions (Lejnine et al., 1995; Wu and de Lange, 2008) and electron microscopy analyses (Nikitina and Woodcock, 2004) indicate that telomeres are organized in a tight nucleosomal array for most of their length. Therefore, it is likely that the structure of telomeric chromatin derives from the interplay between TRF proteins and nucleosomes. *In vitro* studies showed that telomeric proteins such as yeast Rap1 (Rossetti et al., 2001) and human TRF1 (Galati et al., 2006) are able to recognize their binding sites in a nucleosomal context. Nucleosome stability is not affected by the addition of TRF1, even at saturating concentration (Galati et al., 2006); however, TRF1 has the ability to remodel nucleosomes inducing their sliding toward adjacent sequences (Pisano et al., 2010). Other studies examined how TRF2 interacts with telomeric chromatin. Addition of TRF2 to *in vitro* reconstituted telomeric nucleosomal arrays induces their compaction (Baker et al., 2011), whereas TRF1 ability to condense DNA seems inhibited by its acidic N-terminal domain (Poulet et al., 2012). Contrary to this, when added to an *in vitro* chromatin assembly system containing ATP-dependent chromatin remodelers, TRF2 causes nucleosome remodeling by increasing telomeric nucleosomal spacing (Galati et al., 2012). The relationship between telomeric proteins and telomeric nucleosomes has been studied also *in vivo* by altering the expression of shelterin proteins. Deletion of TRF2 or POT1 in mouse embryonic fibroblasts (MEF) leads to telomere deprotection but does not result in any evident alteration of the nucleosomal organization as detected by MNase mapping (Wu and de Lange, 2008). Even depletion of the whole shelterin complex from MEF telomeres does not lead to changes in the MNase profile (Sfeir and de Lange, 2012). These data indicate that the basic organization of telomeric chromatin consists of shortly spaced nucleosomes both in the proximal and in the distal part of telomeres. A different conclusion emerges from a recent paper, in which TRF2 removal from telomeres induced by overexpression of a dominant negative mutant (TRF2^ΔBΔM^) resulted in an increase of histone density at telomeres, as evidenced by ChIP experiments (Galati et al., 2012). The latter result could either derive from an increased accessibility of nucleosomes to anti-histone antibodies in the absence of TRF2 or to the existence of nucleosome-free regions at telomeres not detectable by MNase mapping. Furthermore, it must be considered that telomere structure could be differently organized in the cellular systems used, respectively mouse cells with very long telomeres (about 30 kbp) and human cancer cells with relatively short telomeres (about 7–8 kbp).

The ability of TRF2 to regulate nucleosomal organization at telomeres emerges from experiments in which TRF2 is overexpressed. In mouse keratinocytes, overexpression of TRF2 caused the increase of internucleosomal distance and reduced histone density at telomeres (Benetti et al., 2008b). Enhanced TRF2 expression remodels telomeric nucleosome organization also in human cells from cervix carcinoma and from immortalized fibroblasts, resulting in higher histone density at telomeres along with DNA replication and in increased spacing of telomeric nucleosomes (Galati et al., 2012).

Even if its precise function in telomere regulation remains largely unknown, the interplay of TERRA RNA with telomeric proteins and telomeric chromatin is likely to play an important role. TERRA has been shown to bind directly to TRF1 and TRF2, facilitating the recruitment at telomeres of the heterochromatic proteins ORC (origin recognition protein) and HP1; siRNA depletion of TERRA causes a reduction of heterochromatic marks at telomeres inducing telomere dysfunction (Deng et al., 2009b). In addition, TERRA binding reduces TRF2 ability to condense telomeric DNA (Poulet et al., 2012). Conversely, heterochromatic state influences TERRA expression levels. TERRA transcription depends on subtelomeric DNA methylation (Nergadze et al., 2009), and also on the histone H3K4 methyltransferase MLL (Caslini et al., 2009). Finally, TERRA seems to have a role in the protection of the single-stranded chromosome end by modulating POT1 and RPA1 binding through its interaction with the heterogeneous nuclear ribonucleoprotein A1 (hnRNPA1) (Flynn et al., 2011).

## Conclusion

One of the main unresolved issues in telomere biology concerns the structural changes involved in the switch from a protected to a deprotected state as telomeres shorten. The shelterin complex protects telomeres from the DDR response inhibiting six signaling and repair pathways (Sfeir and de Lange, 2012). Although the generation of ATM-dependent or ATR-dependent DDR at telomeres can be essentially attributed to shelterin removal, there could be different outcomes. The acute deprotection derived from the depletion of shelterin proteins such as TRF2 or POT1 leads to extensive telomere fusions. Instead, the DDR to telomere shortening in human cells is a regulated process resulting in p53-mediated cell cycle arrest and entry in replicative senescence. To explain these differences, a three-state model of telomere protection has been proposed (Cesare et al., 2009; Cesare and Karlseder, 2012). In this model, telomere protection is assured by a closed state characterized by a yet undefined structure that might be represented by the t-loop. Telomere shortening could disrupt this structure leading to an intermediate state recognized as DNA damage, but that retains enough shelterin proteins (particularly TRF2) to prevent NHEJ and thus fusion of chromosome ends. Further shortening results in a fully uncapped state in which the levels of bound shelterin are insufficient to impede end-to-end fusions. Recent observations are consistent with this model. The study of spontaneous telomere deprotection in human primary cells showed that the establishment of DDR at about five telomeres in the absence of end-to-end fusions is required to induce p53-dependent senescence (Kaul et al., 2012). In agreement with the existence of an intermediate state, the analysis of individual telomeres showed that TIFs co-localize with TRF2 and telomeric DNA signals. In addition, the inhibition of p53 leads to senescence bypass and to the appearance of telomere fusions characteristic of a fully uncapped state (Kaul et al., 2012). Further support comes from the observation that DNA damage induced by ionizing radiation in senescent cells is repaired by end-joining at genomic sites but not at telomeres, indicating that TRF2 retains the capacity to prevent NHEJ at telomeres (Fumagalli et al., 2012; Hewitt et al., 2012). Finally, it has been recently shown that the repression of ATM signaling and of NHEJ by TRF2 depends on two different domains of the protein (Okamoto et al., 2013). Using a set of mutant proteins in which TRF2 domains were replaced by the analogous TRF1 domains, the authors demonstrated that the ATM pathway is inhibited by the dimerization domain TRFH, whereas NHEJ repression depends on the C-terminal region of the hinge domain, iDDR, which acts by inhibiting the ubiquitin ligase RNF168, necessary for the recruitment of 53BP1.

**Figure 1:**
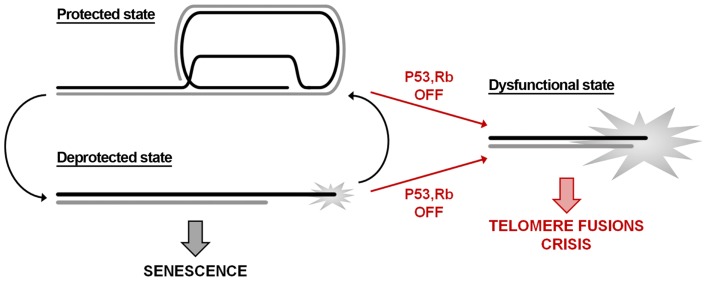
**Telomere state determines cell destiny**. Telomeres swing between a protected and a deprotected state throughout the cell cycle. Accumulation of more than five deprotected telomeres is sufficient to induce replicative senescence. The inactivation of p53/RB signaling pathways allows escaping the growth arrest checkpoint; the consequent further shortening leads to loss of the remaining shelterin and to a dysfunctional state that activates the DNA repair machinery.

In this context, it is worth recalling that cell fate is linked to the stochastic probability of single telomeres to become deprotected (Blackburn, 2000). DNA damage signaling can be found also at long telomeres, although less frequently that at short telomeres (Kaul et al., 2012); in addition, a single deprotected telomere is not sufficient to trigger cell growth arrest, indicating that it might revert to a closed protected state. Indeed, this occurs at every cell cycle: during the S-phase the passage of the replication fork disrupts the closed telomere structure, leading to chromatin rearrangements and to the recruitment of DNA damage factors at telomeres during G2 phase (Verdun et al., 2005). Therefore, in a pre-senescent state, telomeres might switch between a protected and a deprotected state (Figure 1). Telomere erosion eventually shifts the equilibrium toward the deprotected state, leading to cell cycle arrest in G1 and therefore to replicative senescence. Further shortening – for example bypassing growth arrest checkpoint as a result of p53/RB inactivation – irreversibly gives rise to a fully dysfunctional state culminating in telomere fusions and crisis.

**Figure 2:**
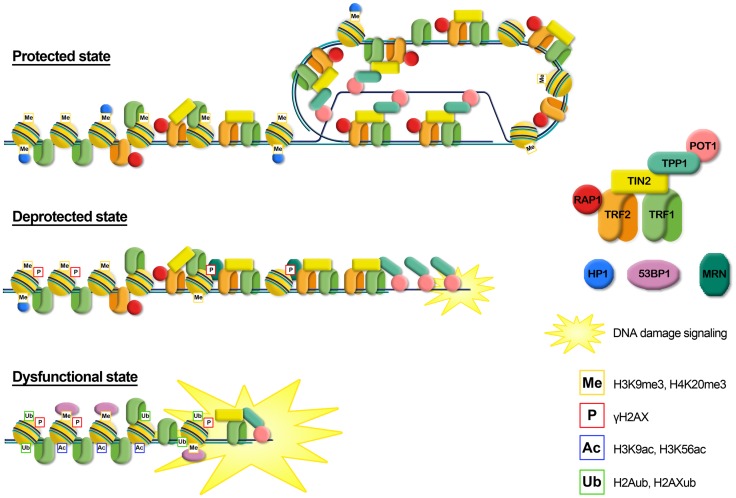
**Graphical representation of the different telomere states, characterized by different levels of telomeric proteins and post-translational modifications**. *Protected state*: telomere is in a closed form, probably the t-loop, maintained by the binding with the shelterin proteins; the presence of trimethylation of histones H3 and H4, typical heterochromatic markers, induces a compacted state. This state inhibits the DNA damage response. *Deprotected state*: telomere shortening could disrupt the closed structure leading to an open state, characterized by a decrease of heterochromatic marks. Telomeres are recognized as DNA damage, signaled by phosphorylation of H2AX, but retain enough shelterin proteins (mainly TRF2) to prevent NHEJ and thus telomeric fusion. DNA damage signaling leads to replicative senescence. *Dysfunctional state*: if growth arrest checkpoint is inactivated, telomeres continue to shorten leading to a fully uncapped form, deriving from the depletion of shelterin proteins such as TRF2 or POT1. Telomere dysfunctions are signaled by phosphorylation of H2AX and the ubiquitylation of H2A and H2AX. Telomeres are not protected from the DNA damage response machinery, giving rise to extensive telomere fusions.

Increasing data are accumulating in support of a role for histone modifications, histone variants, chromatin remodeling complexes and histone chaperones involved in telomere functions and dynamics. Figure 2 summarizes the still incomplete pattern of histone modifications associated with the transition from a protected to a deprotected state and with a fully dysfunctional state. Telomere shortening and the consequent deprotection seem associated with a reduction of heterochromatic marks at telomeres, such as a decrease of H3K9me3 and an increase of acetylated residues. Telomere deprotection and the full uncapping consequent to further shortening are signaled by phosphorylation of H2AX and the ubiquitylation of H2A and H2AX. At present, a clear understanding of the precise timing of appearance of histone modifications and of their functional significance is still lacking. Furthermore, a bias in evaluating the role of histone modifications in the establishment of a protective state at telomeres could derive from the fact that most data on the epigenetic status of telomeres and on telomere deprotection come from studies on murine cells. Lab mice have long telomeres (40–60 kbp) compared to humans (4–15 kbp) and do not undergo cell senescence due to telomere erosion (Itahana et al., 2004; Henriques and Ferreira, 2012). Therefore, extrapolating the data obtained in mice to human telomere could lead to incorrect conclusions.

It seems interesting to highlight that one of the main consequences of nucleosomal organization is to limit the accessibility of telomeric proteins to their binding sites. Both short and long telomeres seem to share the same tight nucleosomal organization that hinders most of the binding sites for TRF1 and TRF2. TRF1 is able to bind quite efficiently also to telomeric repeats on the nucleosome (Galati et al., 2006), whereas the binding of TRF2 at telomeres seems much more hindered by the presence of nucleosomes (Pisano et al., 2008). These differences could partly be attributed to the different N-terminal domains of the two proteins, acidic in the case of TRF1, basic for TRF2; it has recently been proposed that these domains play an important role in regulating telomeric chromatin compaction (Poulet et al., 2012). TRF1 and TRF2 binding in a nucleosomal context could be affected by histone modifications. For example, acetylation of lysine residues reduces the positive charge of histones, increasing the accessibility of nucleosomal DNA to binding proteins (Lee et al., 1993). On the other side, bulky modifications such as ubiquitylation might represent a steric hindrance for the binding of TRF proteins in a nucleosomal context. Therefore, the modifications of histones related with telomere shortening could alter the binding equilibrium of TRF proteins and consequently telomere protection.

The establishment of a mechanism of telomere maintenance is a key factor in the acquisition of the unlimited proliferative capacity of cancer cells (Hanahan and Weinberg, 2011). In this regard, a deeper knowledge of the mechanisms and the structural determinants that regulate telomere stability and control the entry in replicative senescence represents a major goal not only in basic research but also for the implications in the development of anticancer therapies.

## Conflict of Interest Statement

The authors declare that the research was conducted in the absence of any commercial or financial relationships that could be construed as a potential conflict of interest.
